# Treatment of a Type II Endoleak from an Atypical Mediastinal Collateral Branch Following Total Arch Replacement with Frozen Elephant Trunk

**DOI:** 10.3400/avd.cr.25-00124

**Published:** 2026-02-17

**Authors:** Emiko Chiba, Kohei Hamamoto, Mamoru Arakawa, Satoshi Uesugi, Soichiro Kojima, Ryoma Kobayashi, Tatsuya Ogawa, Hiroyuki Fujii, Mitsuru Matsuki, Harushi Mori

**Affiliations:** 1 Department of Radiology, Jichi Medical University, Shimotsuke, Tochigi, Japan; 2 Department of Cardiovascular Surgery, Jichi Medical University, Shimotsuke, Tochigi, Japan

**Keywords:** thoracic artery aneurysm, type II endoleak, transcatheter arterial embolization

## Abstract

We present a rare case of type II endoleak (T2EL) from an atypical mediastinal artery following total arch replacement with a frozen elephant trunk (FET) for chronic aortic dissection. A 63-year-old male with a history of multiple thoracic aortic surgeries, including FET, developed enlargement of an aortic arch aneurysm on follow-up. Computed tomography and diagnostic angiography revealed a T2EL due to a newly developed mediastinal collateral artery arising from the left subclavian artery feeding the sac. Selective transcatheter embolization with N-butyl-2-cyanoacrylate successfully eliminated the endoleak. Recognizing such atypical T2EL sources is crucial for managing post-FET aneurysm expansion.

## Introduction

The hybrid approach for managing thoracic aortic aneurysms using a frozen elephant trunk (FET) has become widely accepted because of its less invasive nature and favorable outcomes.^1)^ A known complication after FET repair is type II endoleak (T2EL), which is typically caused by retrograde flow from aortic branches, such as the intercostal or bronchial arteries, similar to that seen after thoracic endovascular aortic repair (TEVAR).^2,3)^ The arterial pressure in T2EL can lead to aneurysm sac expansion and rupture if left untreated.^4)^ Here, we report a rare case of T2EL originating from an atypical mediastinal collateral artery that was not visualized before FET, developed postoperatively, and was successfully treated with transcatheter arterial embolization (TAE).

## Case Report

**Fig. 1:**
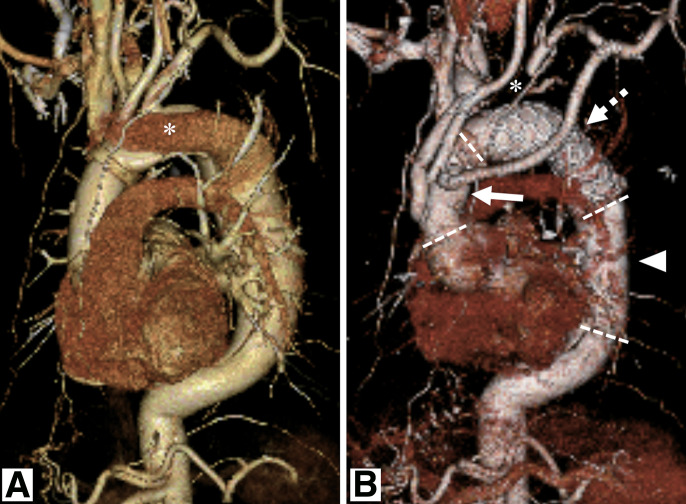
Contrast-enhanced CT images before and after aortic surgery. (**A**) Preoperative VR image prior to total arch replacement with frozen elephant trunk. An asterisk indicates contrast enhancement of the false lumen. (**B**) Postoperative VR image following the final surgery. Arrow: Branched prosthetic graft in the ascending aorta. Dashed arrow: Frozen elephant trunk graft. Arrowhead: Prosthetic graft in the descending aorta. Dashed lines: Anastomosis sites. Note that the left subclavian artery is ligated (*). CT: computed tomography; VR: volume-rendered

**Fig. 2:**
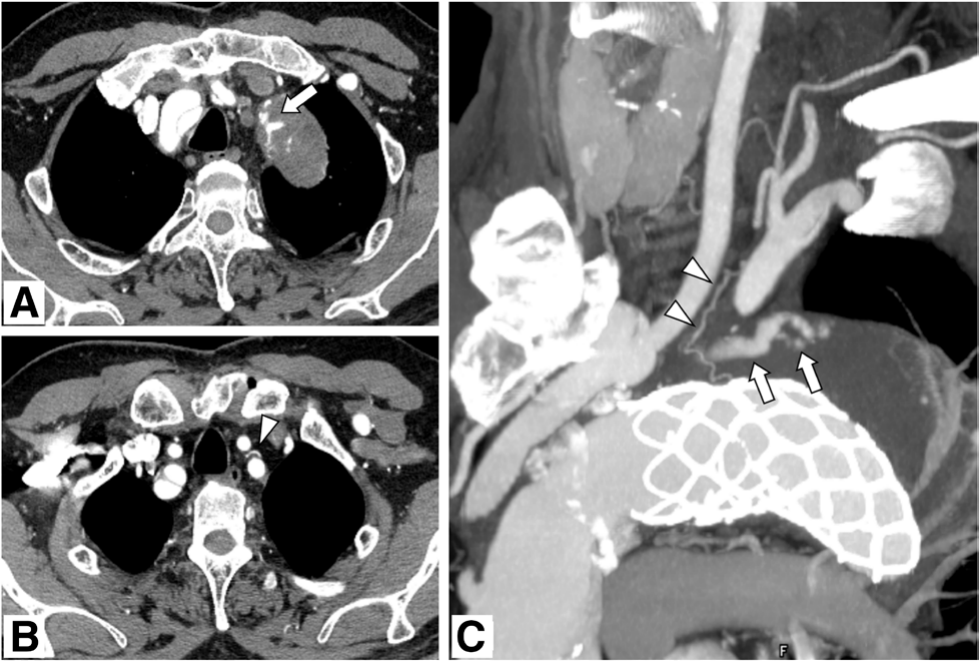
Pre-treatment contrast-enhanced CT. (**A**, **B**) Contrast enhancement within the aneurysm sac suggestive of an endoleak (arrow) and a small mediastinal branch originating from the proximal portion of the left subclavian artery, suspected to be the responsible vessel (arrowhead), is observed. (**C**) Slab MIP image in the left anterior oblique view shows the endoleak within the aneurysm sac (arrows) and the small mediastinal branch (arrowheads). CT: computed tomography; MIP: maximum intensity projection

**Fig. 3:**
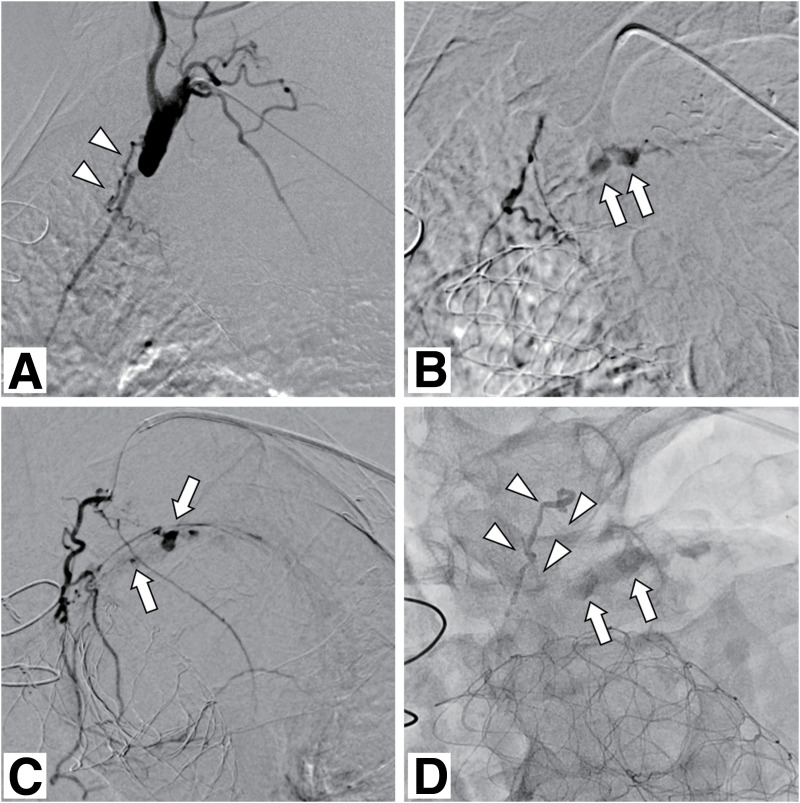
Endovascular treatment. (**A**) Angiography of the left subclavian artery demonstrates a small mediastinal branch corresponding to the findings on contrast-enhanced CT (arrowheads). (**B**, **C**) Selective angiography of the target mediastinal branch in the left anterior oblique (**B**) and anterior (**C**) views shows contrast enhancement within the aneurysm sac via presumed vasa vasorum originating from the distal portion of a small proximally branching vessel. Arrows indicate the endoleak. (**D**) A fluoroscopic image after embolization shows an NBCA cast within the aneurysm sac (arrows) and the target artery (arrowheads). CT: computed tomography; NBCA: N-butyl-2-cyanoacrylate

**Fig. 4:**
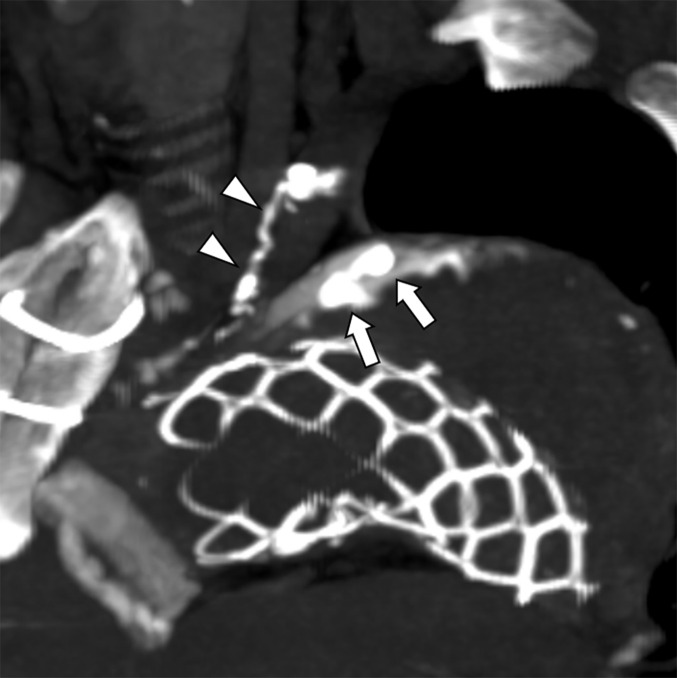
Non-contrast CT after TAE demonstrates deposition of lipiodol within the aneurysm sac (arrows) and the target artery (arrowheads). CT: computed tomography; TAE: transcatheter arterial embolization

A 63-year-old male underwent hemiarch replacement for acute aortic dissection (Stanford type A, DeBakey type I) approximately 13 years earlier. Five years ago, he underwent reoperation consisting of total arch replacement with FET for chronic dissection, together with aortic valve replacement. The distal anastomosis was performed in Zone 1, and the cervical branch ostia were ligated. The left subclavian artery was reconstructed via an extra-anatomic bypass. Three years prior, he underwent descending aortic replacement for residual dissected aorta, with the proximal anastomosis incorporating the distal portion of the FET (**Fig. 1**). His medical history included hyperlipidemia and hypertension. The patient was managed with warfarin therapy following mechanical valve replacement. Contrast-enhanced computed tomography (CT) performed 3 years prior showed no evidence of endoleaks, and the patient was followed up periodically. However, approximately 6 months prior, CT revealed a 6-mm increase in the diameter of the aortic arch aneurysm, with contrast enhancement suggestive of an endoleak. Thin-slice CT revealed a newly developed mediastinal collateral artery originating from the left subclavian artery, which was not observed preoperatively, suggesting a T2EL (**Fig. 2**). Consequently, diagnostic angiography was performed, which confirmed that the collateral artery was the feeding vessel, and the diagnosis of T2EL was established. Following discussions with the cardiovascular surgeon, endovascular treatment was planned, and the patient agreed to this treatment approach. TAE was performed via the left brachial artery access. A 5-Fr guiding sheath (Parent Pulse45; Medikit, Tokyo, Japan) was inserted, and a 4-Fr-shaped catheter (SU-R; Hanaco Medical, Saitama, Japan) was advanced near the target artery. A 1.7-Fr microcatheter (Progreat λ; Terumo, Tokyo, Japan) was coaxially introduced and advanced as distally as possible into the mediastinal collateral artery. Angiography revealed contrast pooling in the aneurysm sac from fine branches, presumed to be vasa vasorum (**Fig. 3A**–**3C**). In addition, an anastomosis with the bronchial artery was visualized in the distal portion of the branch. After confirming the absence of spinal arterial branches, a warm mixture of 14.5% N-butyl-2-cyanoacrylate (NBCA) (B. Braun Aesculap Japan, Tokyo, Japan) and Lipiodol (Guerbet, Villepinte, France) were cautiously injected until it reached the culprit branches and within the aneurysm (**Fig. 3D**). Post-embolization angiography showed the disappearance of the endoleak. Non-contrast CT performed immediately after the procedure confirmed Lipiodol deposition within the aneurysm sac (**Fig. 4**). No intra- or postoperative complications were observed. The patient recovered uneventfully and was discharged on postoperative day 2. Follow-up non-contrast CT at 6 months, 1 year, and 2 months post-TAE revealed no enlargement of the aneurysm sac.

## Discussion

Although T2ELs after FET are relatively rare compared to those following TEVAR, they can still occur and are generally self-limiting.^2,3)^ In cases of T2EL following TEVAR, the bronchial and intercostal arteries are known to be the primary sources. Reported anastomotic branches include the supreme intercostal artery, transverse cervical artery, lateral thoracic artery, and subscapular artery.^3,5–7)^ A similar collateral pathway from branches of the subclavian artery to the descending thoracic aorta, termed mediastinal collateral arteries, has been reported by Kirks et al. in patients with aortic coarctation or subclavian artery obstruction.^8)^ In these cases, the parascapular vessels, including the dorsal scapular, thoracoacromial, lateral thoracic, subscapular, and circumflex scapular arteries, as well as the intercostal and internal thoracic arteries, were identified as major collateral pathways. Although rare, the transverse cervical, carotid, vertebral, and anterior spinal arteries have also been implicated.

In the present case, the responsible vessel for the T2EL was presumed to be the vasa vasorum distributed around the aortic arch or a small branch arising directly from the aortic arch, based on the angiographic findings. Its anastomotic branch arose from a small vessel originating from the proximal subclavian artery. Although the exact anatomical name of this vessel remains unclear, contrast-enhanced CT and angiographic imaging demonstrated that it was distributed in the mediastinum, particularly around the aortic arch and the trachea, and formed an anastomosis with a bronchial artery. These findings suggest that it corresponds to a so-called mediastinal artery or mediastinal branch, which typically arises from the aorta, the internal thoracic artery, or, more rarely, the brachiocephalic artery.^9–11)^ Another possible origin of collateral arteries is the thymic artery, which is known to arise from the internal thoracic artery or the thyrocervical trunk, and rarely from the aorta or the internal carotid artery.^12,13)^ Notably, this vessel in the present case was not visible prior to the FET procedure, suggesting that it may have existed only as a rudimentary microchannel before the procedure and subsequently dilated postoperatively. Furnari et al. reported a tiny anastomosis (shunt) between a bronchial artery and the subclavian artery via a small branch in a patient with cystic fibrosis, suggesting the involvement of a hypertrophied mediastinal artery as a collateral vessel.^14)^ Although the underlying etiology differs, the present case may represent a similar anatomical vulnerability, in which hemodynamic changes following FET led to the dilation of a small subclavian branch, which subsequently became the responsible vessel for the T2EL. To the best of our knowledge, no similar cases have been reported following TEVAR or FET, indicating that this represents a very rare clinical entity. Recognition of such atypical pathways is essential in cases of unexplained sac expansion, and catheter-directed angiography remains valuable for their identification and treatment.

TAE using NBCA was chosen due to its efficacy in achieving rapid and durable occlusion, particularly in small-caliber and tortuous arteries. The procedure resulted in complete resolution of the endoleak and stabilization of the aneurysm sac. Complete embolization of the responsible artery is essential for effective T2EL treatment, and sac embolization has been reported to improve outcomes.^15)^ Although coil embolization may be sufficient when complete occlusion is achievable, proximal embolization with metallic coils or particles often results in incomplete treatment due to the development of collateral pathways, leading to persistent endoleaks. This is of particular concern in cases involving the vasa vasorum, where flow control with coils alone is challenging, and NBCA has been reported to be more effective.^16)^ For these reasons, we used a low-concentration NBCA–lipiodol mixture in the present case. In addition, we heated the NBCA–lipiodol mixture to reduce the viscosity of lipiodol, thereby improving distal penetration and enabling embolization up to the target vessel and into the sac, as previously reported by Nakazawa and Murao.^17)^ However, when embolizing with low-concentration NBCA for lesions with shunting between the target arteries and aortic branches, such as the intercostal artery, it is important to pay attention to unintentional embolization of non-target vessels to avoid serious complications such as spinal paralysis. Therefore, if spinal branches are visualized on angiography, it is necessary to advance the catheter tip beyond these branches before performing embolization. Moreover, because the responsible artery originated from the proximal portion of the subclavian artery, it was essential to prevent glue migration into the subclavian artery to avoid the risk of cerebral infarction. Although direct sac puncture is an alternative treatment option,^6,7)^ it was anatomically challenging in this case, and the small-caliber vessel made selective embolization unfeasible.

## Conclusion

Although mediastinal collateral arteries are rare, they should be considered as potential sources of T2EL after total arch replacement with FET, particularly in cases of unexplained sac enlargement. In such cases, TAE is a feasible and effective treatment option, even in anatomically complex situations. Nevertheless, the long-term outcomes of TAE for T2ELs after thoracic aortic aneurysm repair remain unclear; therefore, continued follow-up is warranted in such cases.
